# Emergence and Characterization of Tigecycline Resistance Gene *tet*(X4) in ST609 Escherichia coli Isolates from Wastewater in Turkey

**DOI:** 10.1128/spectrum.00732-22

**Published:** 2022-07-13

**Authors:** Cemil Kürekci, Xiaoyu Lu, Büsra Gülay Celil, Hüseyin Burak Disli, Mashkoor Mohsin, Zhiqiang Wang, Ruichao Li

**Affiliations:** a Department of Food Hygiene and Technology, Faculty of Veterinary Medicine, Hatay Mustafa Kemal University, Hatay, Turkey; b Jiangsu Co-Innovation Center for Prevention and Control of Important Animal Infectious Diseases and Zoonoses, College of Veterinary Medicine, Yangzhou University, Yangzhou, Jiangsu Province, P. R. China; c Graduate School of Health Sciences, Hatay Mustafa Kemal University, Hatay, Turkey; d Institute of Microbiology, University of Agriculture, Faisalabad, Pakistan; e Institute of Comparative Medicine, Yangzhou University, Yangzhou, Jiangsu Province, P. R. China; Brown University

**Keywords:** *Escherichia coli*, *tet*(X4), wastewater, ST609, *bla*_SHV-12_

## Abstract

Emergence of pathogens harboring tigecycline resistance genes incurs great concerns. Wastewater is recognized as the important reservoir of antimicrobial resistance genes. Here we characterized the phenotypes and genotypes of bacteria carrying *tet*(X4) from wastewater in Turkey for the first time. Four *tet*(X4)-positive Escherichia coli isolates were identified and characterized by PCR, Sanger sequencing, antimicrobial susceptibility testing, conjugation assays, Illumina sequencing, nanopore sequencing and bioinformatic analysis. Four *tet*(X4)-harboring isolates were multidrug-resistant (MDR) bacteria and the *tet*(X4) gene was nontransferable in four isolates. Genetic analysis revealed that *tet*(X4) genes in four isolates were located on plasmids co-harboring two replicons IncFIA(HI1) and IncFIB(K). However, none of the four plasmids carried genes associated with horizontal transfer of plasmids. The coexistence of *bla*_SHV-12_-bearing IncX3-type plasmid and *tet*(X4)-harboring plasmid was also found in one isolate. These findings indicate that continuous surveillance of the *tet*(X4)-bearing isolates in different environments worldwide should be strengthened.

**IMPORTANCE** The emergence of tigecycline resistance genes in humans and animals in China seriously threatens the clinical utility of tigecycline, but the molecular epidemiology of tigecycline-resistant bacteria in other countries remained largely unknown. Therefore, it is necessary to learn the prevalence and molecular characteristics of bacteria carrying tigecycline resistance genes, particularly the mobilizable *tet*(X4), in other countries. In the study, we first described the presence and molecular characteristics of the *tet*(X4)-positive E. coli isolates from wastewater in Turkey. Four *tet*(X4)-bearing isolates belonged to ST609, an E. coli clone commonly found from humans, animals and the environment. These findings highlight the importance of monitoring the *tet*(X4) gene in different settings globally.

## OBSERVATION

Tigecycline is one of the last-resort antimicrobials to treat infections caused by multidrug-resistant (MDR) bacterial pathogens (1, 2). However, mobile tigecycline resistance genes *tet*(X3) and *tet*(X4) were reported in 2019 and aroused worldwide attentions (3, 4). The prevalence of tigecycline resistance genes poses a foreseeable threat to public health. A recent study on tigecycline-resistant E. coli (ST167) occurrence in wastewater has been published from Norway (5). Wastewater is recognized as “hot spots” because of their potential role as the vector and reservoir for pathogens and antimicrobial resistance genes (6, 7). In view of these facts, further studies are warranted to provide insight to understand the role of wastewater for spreading novel tigecycline resistance genes. Here, we first revealed the molecular characteristics of four ST609 E. coli isolates harboring *tet*(X4) from wastewater in Turkey.

A total of 20 wastewater samples, including 10 influent water samples and 10 effluent water samples, were collected from two wastewater treatment plants (Antakya and İskenderun) from March 19, 2021 to May 31, 2021 in Hatay province, Turkey. Four *tet*(X4)-positive isolates, including TKEC21-15 (Antakya effluent water sample), TKEC21-17 (Antakya effluent water sample), TKEC21-42 (İskenderun influent water sample) and TKEC21-59 (Antakya influent water sample), were detected and they were identified as E. coli by 16S rRNA gene sequencing. Antimicrobial susceptibility testing revealed that all four E. coli isolates conferred resistance to tigecycline (Table 1). In addition, they also exhibited resistance to oxytetracycline, tetracycline, doxycycline, ampicillin, amoxicillin and florfenicol (Table 1). TKEC21-17 also conferred resistance to ceftriaxone and ceftiofur. However, conjugation assays showed that *tet*(X4) failed to conjugate into E. coli C600, indicating *tet*(X4) in four isolates were nontransferable.

**TABLE 1:** Antimicrobial susceptibility testing of four *tet*(X4)-harboring isolates against different antimicrobials from Turkey^*a*^

Strain IDs	MICs of antimicrobials (mg/L)																
	TIG	OXY	TET	DOX	AMP	AMX	CEF	CFF	IMP	MEM	STR	GEN	KAN	FFC	CST	ENR	RIF
TKEC21-15	8/R	64/R	32/R	32/R	>128/R	>128/R	≤0.25/S	≤0.25/S	≤0.25/S	≤0.25/S	128/-	1/S	4/S	>128/R	≤0.25/S	1/I	8/-
TKEC21-17	16/R	128/R	128/R	64/R	>128/R	>128/R	16/R	32/R	≤0.25/S	≤0.25/S	>128/-	4/S	8/S	>128/R	≤0.25/S	0.5/I	8/-
TKEC21-42	16/R	>128/R	>128/R	64/R	>128/R	>128/R	≤0.25/S	≤0.25/S	≤0.25/S	0.5/S	128/-	1/S	4/S	>128/R	≤0.25/S	1/I	8/-
TKEC21-59	16/R	128/R	64/R	64/R	>128/R	>128/R	≤0.25/S	1/S	≤0.25/S	≤0.25/S	64/-	2/S	16/S	>128/R	≤0.25/S	1/I	8/-
ATCC 25922	≤0.125	4	0.5	0.5	4	4	≤0.125	≤0.125	≤0.125	≤0.125	4	0.25	2	4	0.25	≤0.125	4

a TIG, tigecycline; OXY, oxytetracycline; TET, tetracycline; DOX, doxycycline; AMP, ampicillin; AMX, amoxicillin; CEF, ceftriaxone; CFF, ceftiofur; IMP, imipenem; MEM, meropenem; STR, streptomycin; GEN, gentamicin; KAN, kanamycin; FFC, florfenicol; CST, colistin; ENR, enrofloxacin; RIF, rifampicin; ATCC, American Type Culture Collection; S, susceptible; I, intermediate; R, resistant; -, no breakpoint was provided.

**FIG 1:**
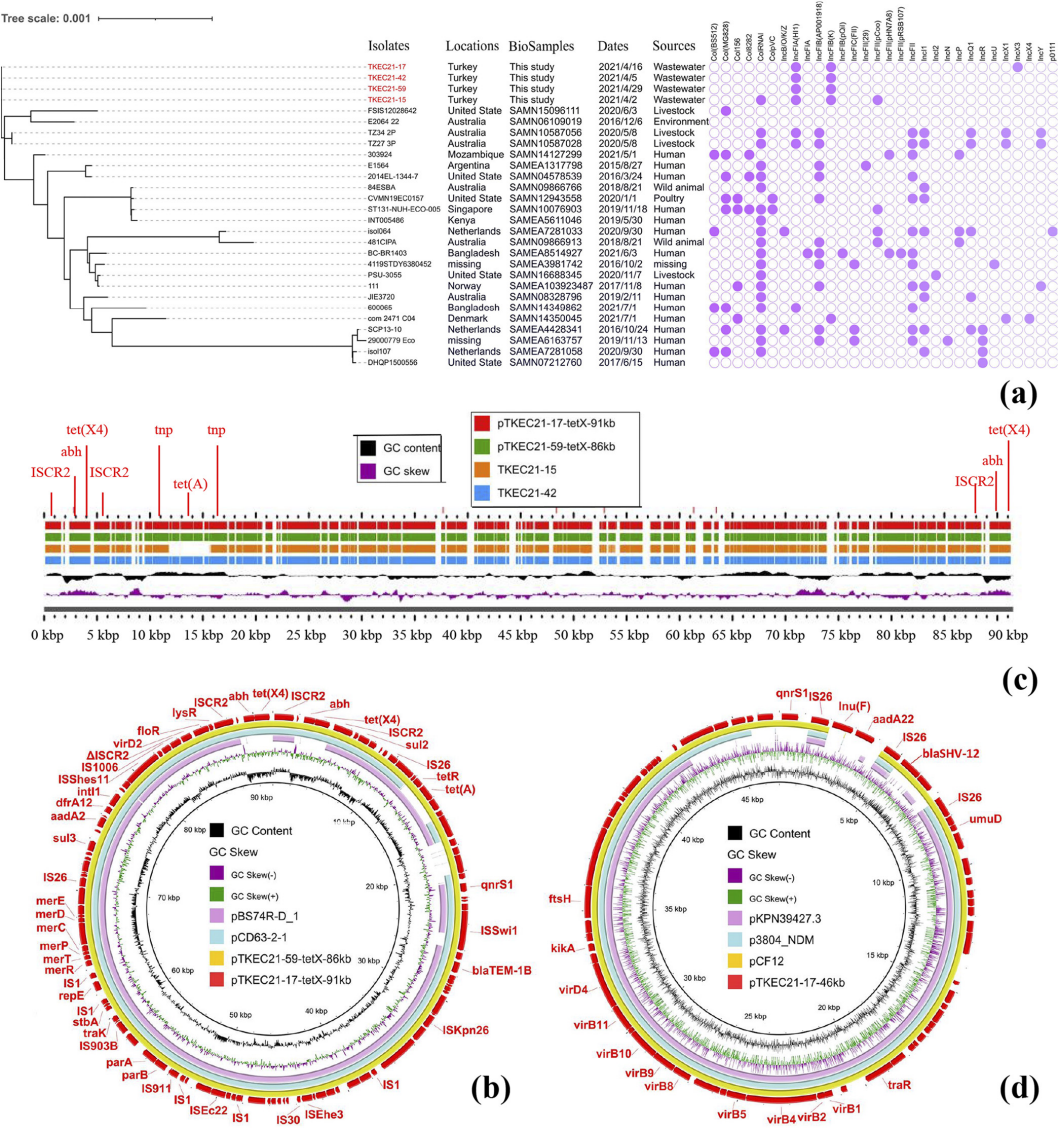
The evolutionary relatedness of the ST609 E. coli isolates and comparison of resistance plasmid structures. (a) Phylogenetic tree of twenty-eight ST609 E. coli isolates including four *tet*(X4)-positive E. coli isolates in this study and twenty-four isolates from the NCBI SRA database. The distribution of replicons was displayed in the figure. The presence or absence of replicons is colored in light purple or light gray, respectively. Phylogenetic tree was visualized by iTOL. (b) Circular comparison of *tet*(X4)-bearing plasmids (pTKEC21-17-tetX-91kb and pTKEC21-59-tetX-86kb) with similar ones in the NCBI database. The outmost circle indicates the plasmid pTKEC21-17-tetX-91kb with genes annotated. (c) Comparison of sequences of *tet*(X4)-bearing plasmids pTKEC21-17-tetX-91kb and pTKEC21-59-tetX-86kb with draft genome sequences of TKEC21-15 and TKEC21-42. (d) Circular comparison of *bla*_SHV-12_-bearing pTKEC21-17-46kb with similar IncX3 plasmids in the NCBI database. The outmost circle indicates the plasmid pTKEC21-17-46kb with genes annotated. Circular comparisons between plasmids were performed using the BRIG. Comparisons between plasmids and draft genome sequences were performed using the website server (https://server.gview.ca/).

Four *tet*(X4)-bearing isolates were performed with whole genome sequencing by Illumina HiSeq 2500 platform, and assembled by SPAdes (8). Multilocus sequence typing (MLST) analysis revealed that four isolates belonged to ST609. TKEC21-42 and TKEC21-59 contained identical antimicrobial resistance genes *tet*(X4), *tet*(A), *bla*_TEM-1B_, *aadA2*, *dfrA12*, *floR*, *sul2*, *sul3* and *qnrS1*. *aadA22*, *bla*_SHV-12_, and *lnu*(F) were additionally found in TKEC21-17 compared to TKEC21-42 and TKEC21-59. All the four *tet*(X4)-bearing isolates carried IncFIA(HI1) and IncFIB(K). TKEC21-17 also contained IncX3, and TKEC21-15 additionally harbored ColRNAI and IncFII(pCoo) (Fig. 1a).

To learn the evolutionary relatedness between four *tet*(X4)-bearing isolates in the study and other twenty-four ST609 E. coli isolates in the NCBI database, a phylogenetic tree based on SNPs of core genomes was constructed using FastTree (9). We found that four *tet*(X4)-bearing isolates from Turkey had large SNP differences with other ST609 E. coli isolates from the NCBI database (Fig. 1a), suggesting that the evolution of ST609 E. coli isolates from Turkey was distinctive. Importantly, we noticed that ST609 E. coli isolates from different countries were mainly from humans (15/24), with few strains from livestock (4/24), wild animals (2/24), poultry (1/24) and the environment (1/24) (Fig. 1a). This implied that ST609 E. coli isolates could spread and evolve in different niches. The emergence of *tet*(X4)-carrying ST609 E. coli isolates poses a challenge to the clinical treatment of carbapenem resistant pathogens. In addition, we noticed that many other types of plasmids can exist in twenty-four ST609 E. coli isolates from the NCBI database (Fig. 1a), such as IncI2 and IncX4 plasmids where *mcr-1* is often located (10), IncQ1 plasmids where *tet*(X4) is often located (11), and IncF plasmids where *bla*_NDM-1_ or *tet*(X4) is often located (12, 13). Among twenty-four ST609 E. coli isolates, isolates 111, 4119STDY6380452 and SCP13-10 carried ΔIS*CR2*, relating to the transfer of *tet*(X4) (3). Therefore, it is also possible that *tet*(X4)-positive ST609 E. coli isolates appear in humans in other countries.

Two representative *tet*(X4)-positive E. coli isolates were selected to perform MinION nanopore sequencing. Unicycler (14, 15) was used for acquiring complete genome sequences. TKEC21-17 harbored a chromosome (4,627,225 bp) and two plasmids pTKEC21-17-tetX-91kb (91,489 bp) and pTKEC21-17-46kb (46,799 bp). The *tet*(X4) gene in E. coli TKEC21-17 was located on the pTKEC21-17-tetX-91kb. A chromosome (4,626,473 bp) and one *tet*(X4)-bearing plasmid pTKEC21-59-tetX-86kb (86,914 bp) were found in TKEC21-59. Both pTKEC21-17-tetX-91kb and pTKEC21-59-tetX-86kb contained IncFIA(HI1) and IncFIB(K). These two plasmids also carried identical resistance genes including *tet*(X4), *tet*(A), *bla*_TEM-1B_, *aadA2*, *dfrA12*, *floR*, *sul2*, *sul3*, and *qnrS1*. BLASTn analysis indicated that pTKEC21-17-tetX-91kb showed 99.87% nucleotide identity at 100% query coverage to pTKEC21-59-tetX-86kb (Fig. 1b). TKEC21-42 and TKEC21-15 also carried IncFIA(HI1) and IncFIB(K) replicons (Fig. 1a). We confirmed that *tet*(X4) in TKEC21-42 and TKEC21-15 were located on IncFIA(HI1)-IncFIB(K) plasmids with similar structures of pTKEC21-17-tetX-91kb and pTKEC21-59-tetX-86kb by comparing draft assembly sequences (Fig. 1c). pTKEC21-17-tetX-91kb exhibited 100% identity at 92% coverage with the plasmid pCD63-2-1 (CP050041) in E. coli 63-2 isolated from broiler chicken in China (Fig. 1b). In addition, pTKEC21-17-tetX-91kb also showed 99.48% identity at 88% coverage to pBS74R-D_1 (CP063333) in E. coli BS74R-D isolated from human in Switzerland (Fig. 1b). Interestingly, two repeats of *tet*(X4) were found in pTKEC21-17-tetX-91kb (Fig. 1b), but only one copy of *tet*(X4) appeared in pTKEC21-59-tetX-86kb. Each *tet*(X4) was flanked by two copies of IS*CR2* and the *tet*(X4)-bearing structure was IS*CR2*-*hp*-*abh*-*tet*(X4)-IS*CR2* with 6,102 bp in length. It has been reported that homologous recombination between two copies of IS*CR2* in the same direction could result in the formation of *tet*(X4)-bearing circular intermediate IS*CR2*-*hp*-*abh*-*tet*(X4) (4,608 bp), which may play an important role in facilitating the transmission of *tet*(X4) (3, 11). None of the four plasmids carried Type IV Secretion System (Fig. 1b), associated with horizontal transfer of plasmids (16). Therefore, *tet*(X4) may be spread by clonal transmission of *tet*(X4)-bearing ST609 E. coli isolates in Turkey.

The *bla*_SHV-12_-bearing pTKEC21-17-46kb coexisted with pTKEC21-17-tetX-91kb. *bla*_SHV-12_ could encode the extended-spectrum beta-lactamase (ESBL) to hydrolyze the β-lactam ring of broad-spectrum β-lactams (17). pTKEC21-17-46kb was an IncX3 plasmid carrying *bla*_SHV-12_, *aadA22*, *qnrS1*, and *lnu*(F). It exhibited 99.99% identity at 95% coverage to *bla*_SHV-12_-bearing pCF12 (MT441556) in Citrobacter freundii CF12 from Spain (Fig. 1d). pTKEC21-17-46kb also showed 99.97% identity at 85% coverage to *bla*_NDM-1_-bearing p3804_NDM (CP064660) in Enterobacter hormaechei 3804 from China and 100% identity at 81% coverage to pKPN39427.3 (CP054267) in Klebsiella pneumoniae 39427 from United States (Fig. 1d). Three similar IncX3 plasmids in *Enterobacteriaceae* strains were isolated from humans. This further highlighted the importance of the epidemic IncX3 plasmids in disseminating resistance genes among humans. IncX3 plasmid was also an important vector of *bla*_NDM-5_ and found frequently in various sources (18, 19). In fact, the coexistence of *bla*_NDM-5_-bearing IncX3 plasmid and *tet*(X4)-harboring plasmid has been found in ST4656 E. coli of animal origin (20). Therefore, the coexistence of *tet*(X4) and *bla*_NDM_ in single isolate from wastewater is also a risk.

In conclusion, we described the emergence and molecular characteristics of *tet*(X4)-positive E. coli isolates from wastewater. Coexistence of *bla*_SHV-12_-bearing IncX3 and *tet*(X4)-harboring plasmids was observed. The coexistence of *tet*(X4) and *bla*_SHV-12_ in single isolate obtained from wastewater may pose a serious public health risk. These findings indicate that continuous surveillance of the *tet*(X4)-bearing isolates and the coexistence of *tet*(X4) and *bla*_SHV-12_ in single bacterial isolates should be strengthened.

### Data availability.

The complete sequences of E. coli TKEC21-17 and TKEC21-59 were deposited in the NCBI database under the following accession numbers: TKEC21-17-chromosome, CP092449; pTKEC21-17-tetX-91kb, CP092450; pTKEC21-17-46kb, CP092451; TKEC21-59-chromosome, CP092452; pTKEC21-59-tetX-86kb, CP092453. The draft genome sequences of E. coli TKEC21-15 and TKEC21-42 could be found in NCBI with the BioProject PRJNA807883.
